# Anlotinib combined with penpulimab in the treatment of advanced adult thoracic spinal osteosarcoma: a case report

**DOI:** 10.3389/fphar.2025.1599496

**Published:** 2025-07-30

**Authors:** Xiaochun Chen, Xianqin Li, Haiying Zhao, Jinwen Qu, Xiaojuan Li, Chuan Lin, Zhiping Yuan

**Affiliations:** Department of Oncology (Radiotherapy), The First People’s Hospital of Yibin, Yibin, Sichuan, China

**Keywords:** spinal osteosarcoma, anlotinib, penpulimab, VEGFr-TKI, anti-PD-1

## Abstract

**Background:**

Spinal osteosarcoma is a rare and prognostically poor subtype of osteosarcoma, with limited efficacy from traditional chemoradiotherapy. The potential of targeted therapy combined with immunotherapy requires further exploration.

**Case Summary:**

A 53-year-old female with stage IV thoracic spinal osteosarcoma initially received intensity-modulated radiotherapy (total dose of 45 Gy in 15 fractions) and AP chemotherapy (doxorubicin 60 mg/m^2^ + cisplatin 100 mg/m^2^). Treatment was discontinued due to grade 4 myelosuppression and sepsis. Subsequently, concurrent combination therapy with anlotinib (12 mg daily for 14 days followed by a 7-day rest) and penpulimab (200 mg intravenously every 3 weeks) was initiated. Penpulimab was administered regularly for 2 years before discontinuation, while anlotinib was reduced to 10 mg daily due to grade 2 hand-foot syndrome and continued thereafter. Post-treatment, the patient achieved significant pain relief, restored self-care capacity, and stable disease (SD) with a progression-free survival (PFS) exceeding 33 months.

**Lessons:**

This case demonstrates that sequential molecular targeted therapy and immunotherapy following chemoradiation can yield remarkable clinical outcomes, offering a novel therapeutic option for advanced spinal osteosarcoma. However, interindividual variations in treatment response underscore the need for future research to identify predictive biomarkers for patient stratification.

## 1 Introduction

Osteosarcoma is a highly aggressive malignant bone tumor that predominantly occurs in adolescents and young adults, but can also affect older patients (Beird et al., 2022). Spinal osteosarcoma accounts for less than 5% of all osteosarcoma cases (Ozaki et al., 2002; Ilaslan et al., 2004). Due to its deep-seated location and proximity to critical neural structures, treatment strategies primarily involving surgery, radiotherapy, and chemotherapy often result in poor prognoses (Wang et al., 2016), with studies reporting a median overall survival of only 23 months (Ozaki et al., 2002).

In recent years, advances in osteosarcoma research have highlighted the growing role of targeted therapy and immunotherapy. Penpulimab is a humanized, high-affinity IgG1 anti-PD-1 monoclonal antibody. Its Fc mutation eliminates Fc receptor and complement-mediated effector functions, likely reducing toxicity (Dahan et al., 2015). This makes it beneficial for advanced/metastatic solid tumors (Zheng et al., 2024). Anlotinib, a novel multi-targeted TKI, has broad-spectrum antitumor activity. It improves the immune microenvironment to inhibit tumor growth (Shen et al., 2018). In recurrent/metastatic osteosarcoma patients treated with anlotinib, studies have reported a 79% disease control rate and a median PFS of 5.3 months (Tang et al., 2020). Anlotinib is now approved in China for osteosarcoma and soft tissue sarcoma treatment and is covered by insurance. In osteosarcoma research, combining anti-PD-1/PD-L1 therapy with anti-angiogenic treatment is gaining more evidence (Yu and Yao, 2024), with multiple studies confirming their synergistic effects.

Additionally, both anlotinib and penpulimab are drugs independently developed and manufactured in China. Due to their low cost (with anlotinib covered by China’s national medical insurance and penpulimab available through a charitable drug donation program) and their remarkable efficacy in cancer treatment, patients in this study ultimately opted for this combination regimen. We detailed the application of this combined therapy in advanced osteosarcoma, aiming to contribute new clinical insights and data to advance therapeutic strategies in this field.

## 2 Case report

### 2.1 Clinical history

**FIGURE 1:**
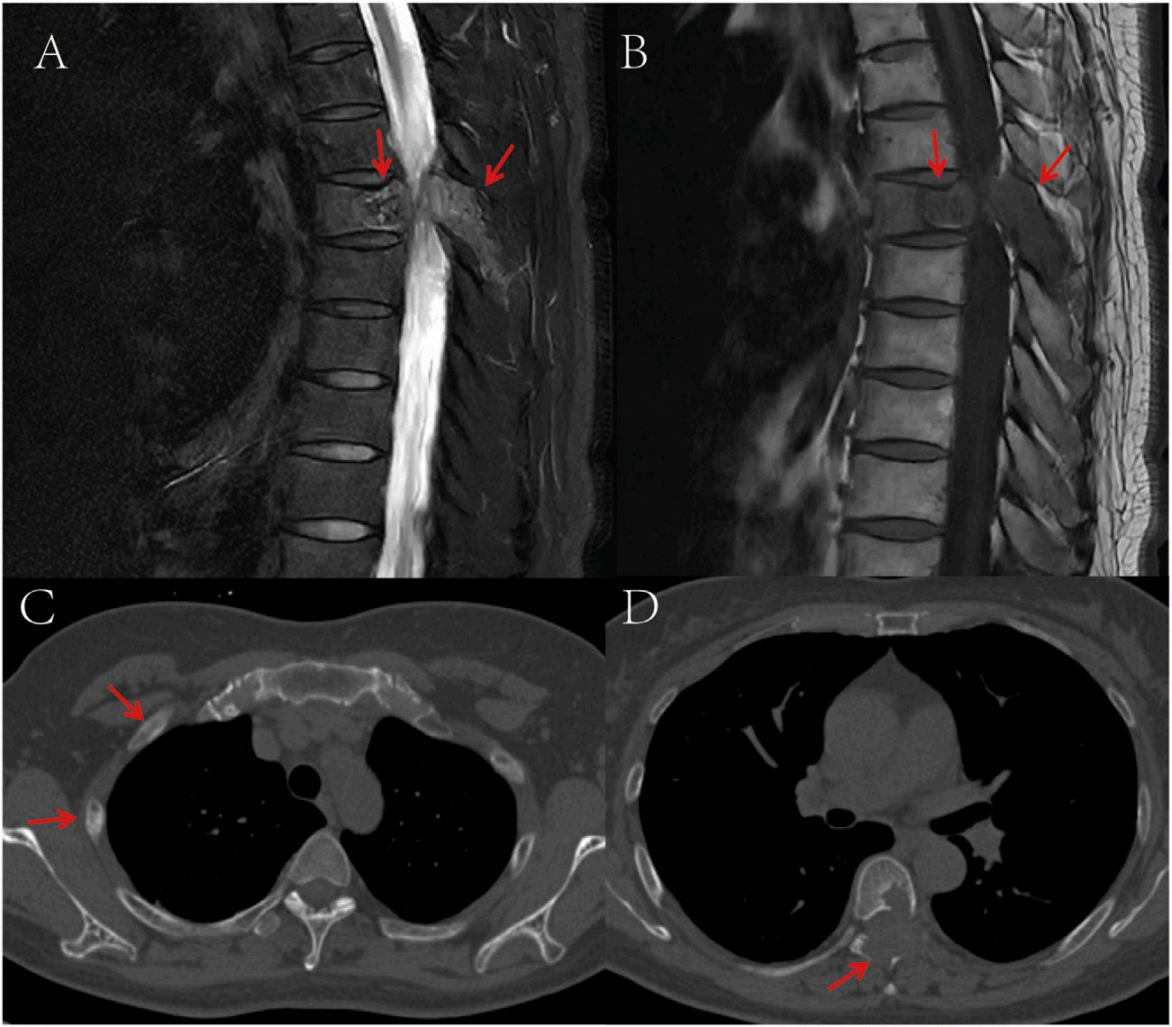
**(A)** T2-weighted MRI shows a space-occupying lesion in the T6 vertebral body and adjacent structures, demonstrating high signal intensity; **(B)** T1-weighted MRI reveals the same T6 vertebral body lesion with low signal intensity; **(C)** CT bone window imaging demonstrates osteoblastic bone destruction in bilateral ribs, suggestive of multiple metastatic lesions; **(D)** Non-contrast CT bone window imaging displays destruction of the T6 vertebral body and adjacent structures accompanied by a soft tissue mass, with punctate and flaky neoplastic bone formation within the lesion. The red arrows in the images indicate the location of the tumor.

**FIGURE 2:**
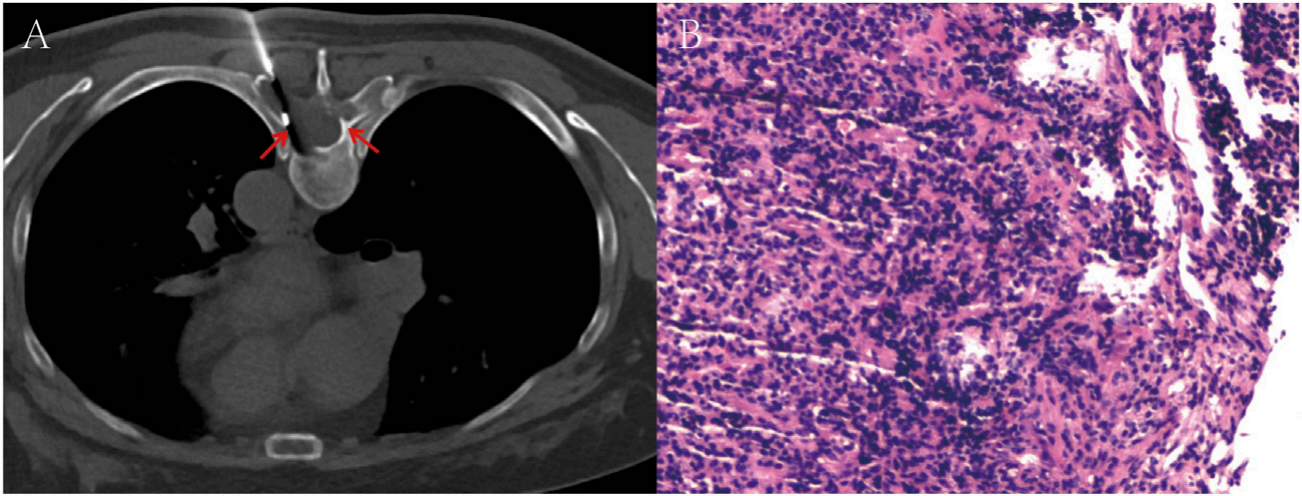
**(A)** Image of a T6 vertebral body soft tissue lesion biopsy performed under local anesthesia. **(B)** Hematoxylin-eosin (H&E) stained section reveals spindle cells and small round cells exhibiting moderate atypia, with focal osteoid formation. The red arrows in the images indicate the location of the tumor.

A 53-year-old female presented with persistent back pain for 3 months and was admitted on 8 March, 2022. Magnetic resonance imaging (MRI) (Figures 1A,B) revealed a neoplastic lesion in the left posterior aspect of the T6 vertebral body and adjacent paravertebral regions, involving the left 5th–7th costovertebral joints, the inferior edge of the T5 spinous process, and the left T6 vertebral appendages. Chest computed tomography (CT) (Figures 1C,D) further demonstrated osteoblastic bone destruction in the left 1st–2nd ribs and right 2nd–4th and 11th ribs, along with osteolytic destruction of the T6 vertebral body and appendages accompanied by soft tissue mass formation and scattered tumor bone formation. A percutaneous biopsy of the thoracic lesion (Figures 2A,B) revealed spindle cells and small round cells with moderate atypia and focal osteoid formation. Immunohistochemistry showed Vimentin (+) and SATB2 (+), confirming the diagnosis of osteosarcoma. Based on imaging and histopathological findings, the patient was diagnosed with stage IV thoracic osteosarcoma (cT1N0M1, AJCC 8th edition).

### 2.2 Laboratory and physical examination

Biochemical tests showed elevated alkaline phosphatase (ALP: 308 U/L) and normal lactate dehydrogenase (LDH: 181 U/L). Liver and renal function, electrolytes, blood glucose, complete blood count, coagulation profile, urinalysis, and tumor markers were within normal ranges. Physical examination revealed an acutely ill appearance with an ECOG performance status (PS) of 1. Tenderness was noted over the T6 spinous process and paravertebral regions, while cardiopulmonary and abdominal examinations were unremarkable.

### 2.3 Initial treatment

From March 17 to 5 May, 2022, the patient underwent intensity-modulated radiotherapy (IMRT) targeting the T6 vertebral lesion, with doses of 45 Gy/15 fractions (3 Gy per fraction) to the gross tumor volume (GTV) and 39 Gy/15 fractions (2.6 Gy per fraction) to the planning target volume (PTV). Concurrently, she received one cycle of chemotherapy with cisplatin, epirubicin, and ifosfamide. Post-chemotherapy, severe myelosuppression developed, including leukopenia (white blood cells: 0.56 × 10^9^/L), neutropenia (absolute neutrophil count: 0.41 × 10^9^/L), and thrombocytopenia (platelets: 18 × 10^9^/L). The patient developed high-grade fever (peak temperature: 41°C) and chills. Inflammatory markers were markedly elevated (IL-6: 3194.6 pg/mL; procalcitonin: 6.656 ng/mL), and blood cultures identified *Streptococcus* pneumoniae, confirming sepsis. Radiotherapy was temporarily suspended. Management included imipenem for infection control, intravenous immunoglobulin, granulocyte colony-stimulating factor (G-CSF), interleukin-11 (IL-11) for platelet recovery, protective isolation, and nutritional support. Five days after infection treatment, the patient’s fever and other symptoms normalized progressively. No further infection signs or symptoms emerged subsequently. To prevent sepsis recurrence, we extended imipenem treatment to a full 14 days course. Radiotherapy was resumed 14 days after interruption.

### 2.4 Subsequent therapy and outcomes

**FIGURE 3:**
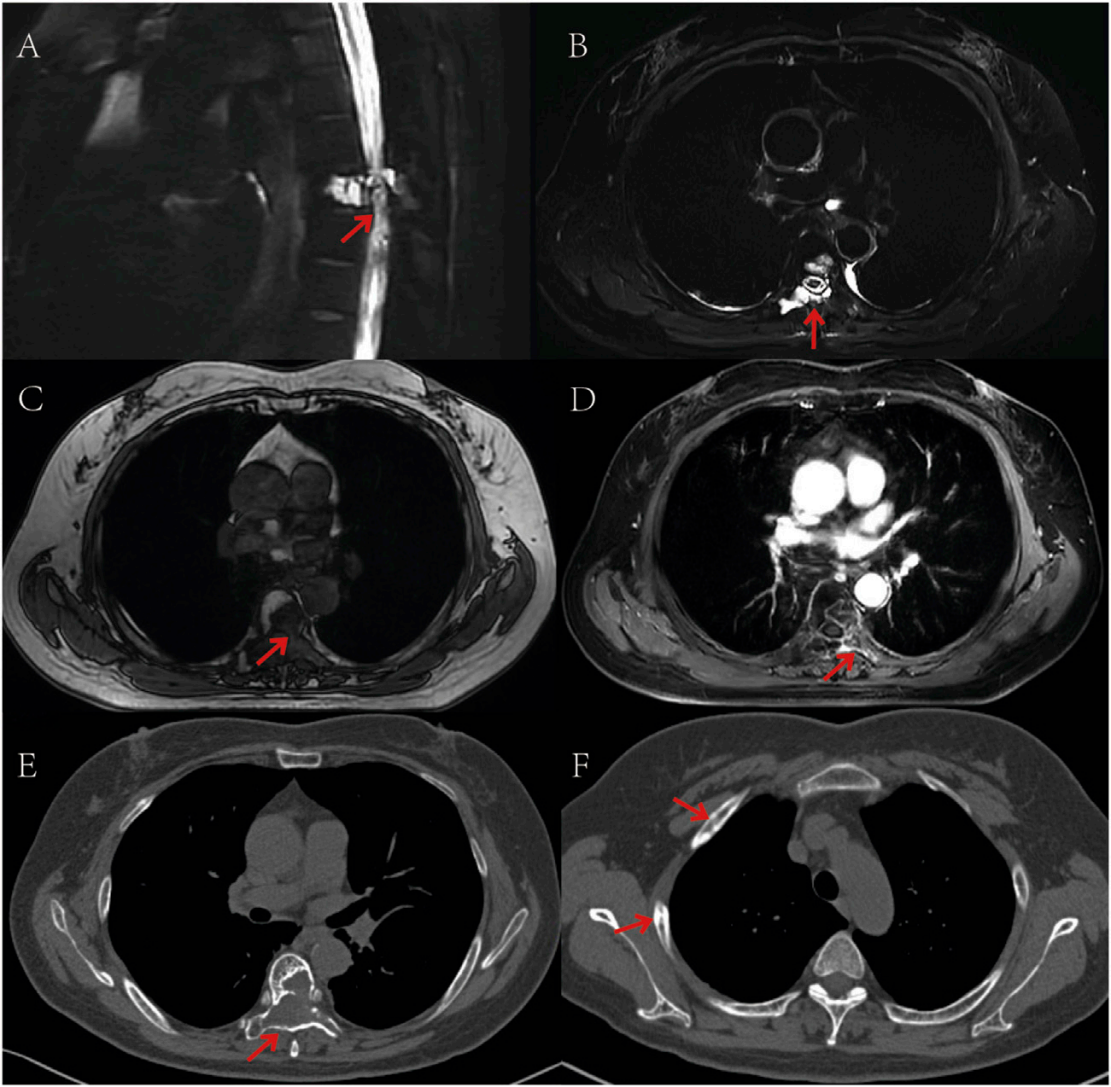
Post-treatment follow-up CT and MR imaging. **(A,B)** T2-weighted MRI sequences demonstrate areas of hyperintense signal corresponding to necrotic-cystic changes; **(C)** Non-contrast T1-weighted MRI sequence reveals a hypointense mass lesion; **(D)** Contrast-enhanced T1-weighted MRI sequence shows peripheral enhancement of the tumor region; **(E)** CT scan (bone window) displays osteolytic destruction of the T6 vertebral body with associated soft tissue mass; **(F)** CT scan (bone window) reveals multiple osteoblastic changes in the ribs. The red arrows in the images indicate the location of the tumor.

On 31 May, 2022, the patient initiated combination therapy with anlotinib (12 mg orally daily for 14 days, followed by a 7-day rest) and penpulimab (200 mg intravenously every 3 weeks). During maintenance therapy, according to the Common Terminology Criteria for Adverse Events version 5.0 (CTCAE 5.0), the patient developed grade 2 hand-foot syndrome, which was intolerable. Consequently, the dose of anlotinib was reduced to 10 mg daily, and in addition, levocetirizine was administered orally and calamine lotion was applied topically. The adjusted dosage was well-tolerated thereafter. No immune-related adverse events (irAEs) have been reported during the penpulimab therapy. Considering the efficacy of anti-PD-1 and the cost burden on the patient, the patient requested to discontinue penpulimab after 2 years of treatment. Currently, the patient reports complete resolution of back pain, restored self-care capacity, and resumed agricultural activities. According to the Response Evaluation Criteria In Solid Tumors version 1.1 (RECIST 1.1), the patient had only one measurable lesion, with a maximum cross-sectional diameter of approximately 3.5 cm. CT or MRI follow-ups were done every 3 months. The target lesion showed partial necrosis but no significant size change, so the treatment response was rated as stable disease (SD) (Figures 3A–F). As of 30 December, 2024, progression-free survival (PFS) has exceeded 33 months.

## 3 Discussion

This patient achieved significant clinical benefits through combination therapy with anlotinib (a VEGFR-TKI) and penpulimab (an anti-PD-1 antibody). Despite experiencing severe myelosuppression and infection during the initial treatment phase, these complications were effectively managed with supportive care, allowing completion of the planned radiotherapy. Subsequent molecular targeted therapy and immunotherapy not only alleviated pain but also restored the patient’s self-care capacity. The treatment response was evaluated as stable disease (SD), indicating effective disease control.

Spinal osteosarcoma can arise in any spinal segment. A study of 198 patients demonstrated that most tumors originate from posterior elements with partial vertebral involvement, while purely intravertebral lesions are rare, with thoracic spine involvement being most common (Ilaslan et al., 2004). The predominant clinical manifestation is insidious back pain, with neurological symptoms emerging only when nerve root compression or spinal cord invasion occurs (Katonis et al., 2013). Notably, 92% of patients present with pain at diagnosis (Groves et al., 2015), consistent with this case characterized by back pain without neurological deficits.

Imaging features on CT typically include osteoid matrix deposition, eccentric osteolytic destruction with patchy tumor bone formation, and paravertebral soft tissue masses (Ropper et al., 2012; Ariyaratne et al., 2023). MRI findings often reveal hypointense or mixed hypointense-isointense signals on T1-weighted imaging and isointense or mixed isointense-hyperintense signals on T2-weighted imaging, with heterogeneous or peripheral enhancement post-contrast (Ropper et al., 2012; Ariyaratne et al., 2023). The current case exhibited eccentric osteolysis, soft tissue mass, and scattered tumor bone formation, aligning with these reported characteristics.

Conventional treatment strategies (surgery, radiotherapy, and chemotherapy) yield suboptimal outcomes. Schoenfeld et al. (Schoenfeld et al., 2010) reported a median overall survival (OS) of 29.5 months in 26 patients, of whom only 10 were metastasis-free at baseline; seven underwent *en bloc* resection, 24 received no radiotherapy, and 25 had no chemotherapy. Similarly, as reported by Evenhuis et al., 40% of patients exhibit a favorable histological response to chemotherapy, which is associated with a better prognosis (Evenhuis et al., 2021). However, in this study, the patient developed grade IV myelosuppression and sepsis after chemotherapy and was considered intolerant to further chemotherapy. The radiotherapy regimen for this patient was based on the study by Boyce-Fappiano et al., which involved administering 45 Gy in 15 fractions to 73 consecutive sarcoma patients, with most tumors located in the trunk (n = 48, 66%). The study showed that 95% of patients experienced symptom relief (Boyce-Fappiano et al., 2022). Radiotherapy is known to enhance systemic immune responses and induce the abscopal effect, where tumors outside the irradiated area also regress, which is particularly useful for optimizing the potential of immunotherapy, especially for bone metastases (Cornillon et al., 2024). Therefore, for palliative care in advanced spinal osteosarcoma patients, especially those with pain, radiotherapy combined with systemic therapy may improve outcomes. However, more evidence is needed to support this approach.

Recent advances in targeted and immunotherapies have reshaped osteosarcoma management. VEGFR-TKIs (e.g., apatinib, anlotinib, sunitinib) inhibit tumor proliferation, metastasis, and angiogenesis by promoting apoptosis (Wilhelm et al., 2004; Liu et al., 2017; Wang et al., 2019; Duan et al., 2020). Study on anlotinib monotherapy for recurrent/metastatic osteosarcoma has reported a median PFS of 5.3 months^8^. Furthermore, multiple studies confirm that combining VEGFR-TKIs with chemotherapy enhances efficacy in advanced/metastatic osteosarcoma. Lenvatinib with etoposide plus ifosfamide in patients with refractory or relapsed osteosarcoma have reported the PFS at 4 months was 51% (Gaspar et al., 2021). Lenvatinib Plus Ifosfamide and Etoposide in Children and Young Adults With Relapsed Osteosarcoma have reported the median PFS was 6.5 months (Xie et al., 2021). Apatinib plus ifosfamide and etoposide for relapsed or refractory osteosarcoma have reported the median event-free survival was 11.4 months (Gaspar et al., 2024). Immune checkpoint inhibitors (ICIs) represent another breakthrough, as antiangiogenic agents remodel the tumor immune microenvironment (TIME) by increasing immune cell infiltration and modulating PD-L1 expression, thereby synergizing with ICIs (Zheng et al., 2018; Liu et al., 2020). Apatinib plus camrelizumab for advanced osteosarcoma reported the 6-month PFS rate was 50.9% (Xie et al., 2020). Camrelizumab and apatinib for advanced bone and soft-tissue sarcomas reported ths PFS was 7.7 months (Zhou et al., 2023). Nivolumab and sunitinib combination for advanced soft tissue sarcomas reported the 6-month PFS rate was 48% (Martin-Broto et al., 2020). In this case, the combination of anlotinib and penpulimab post-palliative chemoradiation resulted in a progression-free survival exceeding 33 months, highlighting its therapeutic potential. In the meantime, the combination of anlotinib and penpulimab has also demonstrated remarkable efficacy in other solid tumor settings. This combination has shown significant efficacy in treating recurrent/metastatic cervical cancer, with an mPFS of 11.0 months (Zhou et al., 2025). In advanced hepatocellular carcinoma, it also demonstrated a significant improvement in median progression-free survival compared to sorafenib (6.9 months vs. 2.8 months) (Wang et al., 2025).

Moreover, in this study, the patient developed an intolerable hand-foot syndrome during anlotinib treatment, leading to a dose reduction from 12 mg to 10 mg. After a literature review, a study was found reporting that the median PFS for advanced sarcoma patients with anlotinib dose reduction and no dose reduction were 8.20 and 6.70 months respectively, with no statistical difference (P = 0.972). However, the median PFS for four patients with hand-foot syndrome was 14.10 months, longer than the 6.00 months for those without (P = 0.024) (Yan et al., 2023). Therefore, proper anlotinib dose adjustment when patients have intolerable adverse reactions may be a good choice.

This study also highlights a discrepancy in treatment response evaluation. The patient’s pain resolved, and self-care ability recovered, but per RECIST 1.1, the response was SD due to no significant lesion size change despite necrosis. However, could this situation be considered as a partial response (PR)? In hepatocellular carcinoma, the mRECIST criteria resolve this by measuring only arterial-enhancing lesions (Llovet and Lencioni, 2020). Future research could explore whether a similar approach applies to osteosarcoma, especially spinal cases, by investigating the proportion of patients with post-treatment tumor necrosis without size change.

Although our patient responded robustly to combination therapy, interpatient variability necessitates biomarker-driven stratification. Examples of such biomarkers include PD-L1 expression, Tumor Mutation Burden (TMB), Tumor Infiltrating Lymphocyte (TIL) density, and VEGF pathway markers to identify optimal candidates for such regimens. A limitation of this study is the absence of supporting data to elucidate the synergistic mechanism of the combined treatment for Spinal Osteosarcoma.

## 4 Conclusion

In this case report, a 53-year-old female with stage IV thoracic osteosarcoma achieved marked clinical efficacy through combination therapy with anlotinib (a VEGFR-TKI) and penpulimab (an anti-PD-1 antibody). Despite severe myelosuppression and infection during initial treatment, subsequent molecular targeted therapy and immunotherapy not only significantly alleviated pain but also restored the patient’s self-care capacity. The treatment response was evaluated as stable disease (SD), with a progression-free survival (PFS) exceeding 33 months. This regimen provides a novel therapeutic option for advanced osteosarcoma patients, demonstrating significant clinical implications. In the future, initiating phase II trials of anlotinib plus anti-PD-1 therapy for spinal osteosarcoma with mandatory biomarker profiling is warranted.

## Data Availability

The original contributions presented in the study are included in the article/supplementary material, further inquiries can be directed to the corresponding authors.

## References

[B1] Ariyaratne S. Jenko N. Iyengar K. P. James S. Mehta J. Botchu R. (2023). Primary osseous malignancies of the spine. Diagn. (Basel) 13 (10), 1801. 10.3390/diagnostics13101801 PMC10217758 37238285

[B2] Beird H. C. Bielack S. S. Flanagan A. M. Gill J. Heymann D. Janeway K. A. (2022). Osteosarcoma. Nat. Rev. Dis. Prim. 8 (1), 77. 10.1038/s41572-022-00409-y 36481668

[B3] Boyce-Fappiano D. Damron E. P. Farooqi A. Mitra D. Conley A. P. Somaiah N. (2022). Hypofractionated radiation therapy for unresectable or metastatic sarcoma lesions. Adv. Radiat. Oncol. 7, 100913. 10.1016/j.adro.2022.100913 35647398 PMC9133361

[B34] Cornillon P. Bouleftour W. Reynaud T. Pigne G. Maillet D. Hamizi S. (2024). Immunogenicity of radiotherapy on bone metastases from prostate adenocarcinoma: What is the future for the combination with radiotherapy/immunotherapy? Tumori. 110, 319–326. 10.1177/03008916241249366 38745528

[B35] Dahan R. Sega E. Engelhardt J. Selby M. Korman A. J. Ravetch J. V. (2015). cγRs modulate the anti-tumor activity of antibodies targeting the PD-1/PD-L1 axis. Cancer Cell 28, 285–295. 10.1016/j.ccell.2015.08.004 26373277

[B4] Duan X. L. Guo J. P. Li F. Xiu C. Wang H. (2020). Sunitinib inhibits PD-L1 expression in osteosarcoma by targeting STAT3 and remodels the immune system in tumor-bearing mice. Future Oncol. 16 (24), 1815–1824. 10.2217/fon-2019-0725 32511016

[B5] Evenhuis R. E. Acem I. Rueten-Budde A. J. Karis D. S. A. Fiocco M. Dorleijn D. M. J. (2021). Survival analysis of 3 different age groups and prognostic factors among 402 patients with skeletal high-grade osteosarcoma. Real world data from a single tertiary sarcoma center. Cancers (Basel) 13, 486. 10.3390/cancers13030486 33513855 PMC7865349

[B6] Gaspar N. Hung G. Y. Strauss S. J. Campbell-Hewson Q. Dela Cruz F. S. Glade Bender J. L. (2024). Lenvatinib plus ifosfamide and etoposide in children and young adults with relapsed osteosarcoma: a phase 2 randomized clinical trial. JAMA Oncol. 10 (12), 1645–1653. 10.1001/jamaoncol.2024.4381 39418029 PMC11581622

[B7] Gaspar N. Venkatramani R. Hecker-Nolting S. Melcon S. G. Locatelli F. Bautista F. (2021). Lenvatinib with etoposide plus ifosfamide in patients with refractory or relapsed osteosarcoma (ITCC-050): a multicentre, open-label, multicohort, phase 1/2 study. Lancet Oncol. 22 (9), 1312–1321. 10.1016/S1470-2045(21)00387-9 34416158

[B8] Groves M. L. Zadnik P. L. Kaloostian P. Sui J. Goodwin C. R. Wolinsky J. P. (2015). Epidemiologic, functional, and oncologic outcome analysis of spinal sarcomas treated surgically at a single institution over 10 years. Spine J. 15 (1), 110–114. 10.1016/j.spinee.2014.07.005 25041727

[B10] Ilaslan H. Sundaram M. Unni K. K. Shives T. C. (2004). Primary vertebral osteosarcoma: imaging findings. Radiology 230 (3), 697–702. 10.1148/radiol.2303030226 14749514

[B11] Katonis P. Datsis G. Karantanas A. Kampouroglou A. Lianoudakis S. Licoudis S. (2013). Spinal osteosarcoma.Clin. Med. Insights Oncol. 7, 199–208. 10.4137/CMO.S10099 24179411 PMC3813616

[B13] Liu K. Ren T. Huang Y. Sun K. Bao X. Wang S. (2017). Apatinib promotes autophagy and apoptosis through VEGFR2/STAT3/BCL-2 signaling in osteosarcoma. Cell Death Dis. 8 (8), e3015. 10.1038/cddis.2017.422 28837148 PMC5596600

[B14] Liu S. Qin T. Liu Z. Wang J. Jia Y. Feng Y. (2020). Anlotinib alters tumor immune microenvironment by downregulating PD-L1 expression on vascular endothelial cells. Cell Death Dis. 11 (5), 309. 10.1038/s41419-020-2511-3 32366856 PMC7198575

[B15] Llovet J. M. Lencioni R. (2020). mRECIST for HCC: Performance and novel refinements. J. Hepatol. 72, 288–306. 10.1016/j.jhep.2019.09.026 31954493 PMC12452114

[B16] Martin-Broto J. Hindi N. Grignani G. Martinez-Trufero J. Redondo A. Valverde C. (2020). Nivolumab and sunitinib combination in advanced soft tissue sarcomas: a multicenter, single-arm, phase Ib/II trial. J. Immunother. Cancer 8 (2), e001561. 10.1136/jitc-2020-001561 33203665 PMC7674086

[B17] Ozaki T. Flege S. Liljenqvist U. Hillmann A. Delling G. Salzer-Kuntschik M. (2002). Osteosarcoma of the spine: experience of the cooperative osteosarcoma study group. Cancer 94 (4), 1069–1077. 10.1002/cncr.10258 11920477

[B18] Ropper A. E. Cahill K. S. Hanna J. W. McCarthy E. F. Gokaslan Z. L. Chi J. H. (2012). Primary vertebral tumors: a review of epidemiologic, histological and imaging findings, part II: locally aggressive and malignant tumors. Neurosurgery 70 (1), 211–219. 10.1227/NEU.0b013e31822d5f17 21768918

[B19] Schoenfeld A. J. Hornicek F. J. Pedlow F. X. Kobayashi W. Garcia R. T. DeLaney T. F. (2010). Osteosarcoma of the spine: experience in 26 patients treated at the Massachusetts general hospital. Spine J. 10 (8), 708–714. 10.1016/j.spinee.2010.05.017 20650409

[B31] Shen G. Zheng F. Ren D. Du F. Dong Q. Wang Z. (2018). Anlotinib: a novel multi-targeting tyrosine kinase inhibitor in clinical development. J. Hematol. Oncol. 11, 120. 10.1186/s13045-018-0664-7 30231931 PMC6146601

[B32] Tang L. Niu X. Wang Z. Cai Q. Tu C. Fan Z. (2020). A phase II study of anlotinib in treating patients with relapsed or metastatic primary malignant bone tumor. JCO 38, 11525–11525. 10.1200/JCO.2020.38.15_suppl.11525

[B33] Wang D. Liu H. Chen L. He M. Zhao W. Xiang Y. (2025). A phase II study of anlotinib plus penpulimab as first‐line treatment for persistent, recurrent, or metastatic cervical cancer: Results from ALTER‐GO‐020 trial. JCO 43, 5527–5527. 10.1200/JCO.2025.43.16_suppl.5527

[B20] Wang G. Sun M. Jiang Y. Zhang T. Sun W. Wang H. (2019). Anlotinib, a novel small molecular tyrosine kinase inhibitor, suppresses growth and metastasis *via* dual blockade of VEGFR2 and MET in osteosarcoma. Int. J. Cancer 145 (4), 979–993. 10.1002/ijc.32180 30719715

[B21] Wang H. Goldberg S. Stanton T. Harmon D. Choy E. Cote G. (2016). Osteosarcoma of the spine and pelvis: one hundred fifteen patients of a single institution. Int. J. Radiat. Oncol. Biol. Phys. 96 (5), E708. 10.1016/j.ijrobp.2016.06.2401

[B22] Wilhelm S. M. Carter C. Tang L. Wilkie D. McNabola A. Rong H. (2004). BAY 43-9006 exhibits broad spectrum oral antitumor activity and targets the RAF/MEK/ERK pathway and receptor tyrosine kinases involved in tumor progression and angiogenesis. Cancer Res. 64 (19), 7099–7109. 10.1158/0008-5472.CAN-04-1443 15466206

[B23] Xie L. Xu J. Sun X. Guo W. Gu J. Liu K. (2020). Apatinib plus camrelizumab (anti-PD1 therapy, SHR-1210) for advanced osteosarcoma (APFAO) progressing after chemotherapy: a single-arm, open-label, phase 2 trial. J. Immunother. Cancer 8 (1), e000798. 10.1136/jitc-2020-000798 32376724 PMC7223462

[B24] Xie L. Xu J. Sun X. Li X. Liu K. Liang X. (2021). Apatinib plus ifosfamide and etoposide for relapsed or refractory osteosarcoma: a retrospective study in two centres. Oncol. Lett. 22 (1), 552. 10.3892/ol.2021.12813 34093773 PMC8170178

[B25] Yan Q. Yao W. T. Du X. H. Guo L. Y. Fan Y. C. (2023). Efficacy and safety of Anlotinib in the treatment of advanced sarcoma. Zhonghua Zhong Liu Za Zhi 45, 904–910. 10.3760/cma.j.cn112152-20210820-00632 37875427

[B30] Yu S. Yao X. (2024). Advances on immunotherapy for osteosarcoma. Mol. Cancer. 23, 192. 10.1186/s12943-024-02105-9 39245737 PMC11382402

[B26] Zheng B. Ren T. Huang Y. Guo W. (2018). Apatinib inhibits migration and invasion as well as PD-L1 expression in osteosarcoma by targeting STAT3. Biochem. Biophys. Res. Commun. 495 (2), 1695–1701. 10.1016/j.bbrc.2017.12.032 29225166

[B28] Zheng Y. Zhu J. Xiong J. Jiang O. Wang H. Xie Y. (2024). Phase 1b/2 study of penpulimab (AK105), an antiprogrammed cell death-1 immunoglobulin G1 antibody, in advanced or metastatic solid tumors (AK105-204). Cancer 130, 2180–2190. 10.1002/cncr.35259 38412283

[B29] Zhou J. Bai L. Luo J. Bai Y. Pan Y. Yang X. (2025). Anlotinib plus penpulimab versus sorafenib in the first-line treatment of unresectable hepatocellular carcinoma (APOLLO): a randomised, controlled, phase 3 trial. Lancet Oncol. 26, 719–731. 10.1016/S1470-2045(25)00190-1 40349716

[B27] Zhou Y. Li M. Zhang B. Yang C. Wang Y. Zheng S. (2023). A pilot study of multi-antigen stimulated cell therapy-I plus camrelizumab and apatinib in patients with advanced bone and soft-tissue sarcomas. BMC Med. 21 (1), 470. 10.1186/s12916-023-03132-x 38031088 PMC10687909

